# The effects of nanocavity and photonic crystal in InGaN/GaN nanorod LED arrays

**DOI:** 10.1186/s11671-016-1548-9

**Published:** 2016-07-20

**Authors:** Qianqian Jiao, Zhizhong Chen, Yulong Feng, Shunfeng Li, Shengxiang Jiang, Junze Li, Yifan Chen, Tongjun Yu, Xiangning Kang, Bo Shen, Guoyi Zhang

**Affiliations:** State Key Laboratory for Artificial Microstructure and Mesoscopic Physics, School of Physics, Peking University, Haidian, Beijing China; Dongguan Institute of Optoelectronics, Peking University, Dongguan, 523808 Guangdong China

**Keywords:** GaN, Light emitting diode, Nanocavity, Photonic crystal, Guided modes

## Abstract

InGaN/GaN nanorod light-emitting diode (LED) arrays were fabricated using nanoimprint and reactive ion etching. The diameters of the nanorods range from 120 to 300 nm. The integral photoluminescence (PL) intensity for 120 nm nanorod LED array is enhanced as 13 times compared to that of the planar one. In angular-resolved PL (ARPL) measurements, there are some strong lobes as resonant regime appeared in the far-field radiation patterns of small size nanorod array, in which the PL spectra are sharp and intense. The PL lifetime for resonant regime is 0.088 ns, which is 40 % lower than that of non-resonant regime for 120 nm nanorod LED array. At last, three dimension finite difference time domain (FDTD) simulation is performed. The effects of guided modes coupling in nanocavity and extraction by photonic crystals are explored.

## Background

Nanoscale light-emitting devices have attracted much attention for their potential applications in biotechnology [1, 2], communication [3] and solid state lighting [4, 5]. Compared to planar light-emitting diodes (LEDs), nanorod LEDs show high performances with higher internal quantum efficiency (IQE), higher light extraction efficiency (LEE) and optimal directionality [4–20]. The improvement of IQE is reasonable for nanorod LEDs, because of the strain relaxation [6–9] and extra in-plane excitonic confinement [7, 10] in InGaN active layer. Moreover, the nanocavity effect is confirmed to enhance the spontaneous emission (SpE) rate in well-ordered nanostructures [11, 12]. Nevertheless, the emission intensity of nanorod array is improved by an order of magnitude or more, which is mainly due to the reduction of modified guided modes [13–15]. Kuo et al [13] reported an ultrahigh extraction efficiency of 79 % at *λ* = 460 nm for a 100-nm diameter nanorod LED without packaging. Three key mechanisms are suggested for the high efficiency: guided modes reduction, embedded quantum wells (QWs) and ultra-efficient out-coupling of fundamental modes. Furthermore, the radiation patterns can be controlled when the guided modes are modified by nanostructures [8, 13, 16–18]. However, the mechanism of light emitting from nanorod array, including light extraction from the nanocavity and light diffraction by their array, is not clear yet. No vertical index confinement makes the mode distribution more complex [19]. Although thermal dissipation and defects/surface states should be dealt with for practical applications [8, 20], the optical modes in nanorod LEDs array are worthy of being manipulated exactly and carefully.

The luminescence lifetimes of nanorod LEDs have been reported by some groups [7, 8, 10–12, 21, 22]. The radiative recombination rate is enhanced when the size of nanorod decreases [14, 21]. It is due to the reduction of the quantum confined Stark effect (QCSE) caused by the strain relaxation in InGaN QWs. However, photoluminescence (PL) decay time for nanorod LEDs may be much longer than that of the planar one [7, 8, 10, 22]. The causes include long exciton diffusion length [7], deep localization in the band-tail [10] and surface localization [22], and so on. It is well known that the SpE is inhibited in photonic bandgap (PBG) [23]. The propagation modes in the photonic crystal (PhC) do not alter SpE significantly [19]. On the other hand, the strong coupling of quantized photon modes with quantized excitations in confined photon structure will enhance SpE rate about two orders [11, 12]. The blue stimulated emission has been obtained by coupling a specific mode with InGaN active layer in nanocavity [24]. However, the contribution of optical modes is seldom distinguished from SpE rate in nanorod LED [25].

The dry etching damage control is also important for nanorod LED. High temperature annealing or wet etching can be used to recover or remove the several tens nm defective layer [16, 20, 26]. Furthermore, the wet etching is suitable for fabricating straight, smooth and well faceted nanorods [20]. Due to the difference in surface potential between n-type and p-type GaN, “flashlight” shaped nanorod can be easily obtained by specific wet etching. The “flashlight” shaped nanorod shows a bottleneck in the region of MQWs layer and n-GaN layer, which would reduce guided modes and enhance the light emitting out of the sidewall. Then, the light into the bottom layer would be reduced.

In this work, the “flashlight” shaped nanorod arrays with top diameters from 120 to 300 nm were fabricated by nanoimprint, induced coupled plasma (ICP) etching and KOH wet etching. Temperature-dependent PL (TDPL), time-resolved PL (TRPL), and angular-resolved PL (ARPL) spectra were measured to study on the effects of nanocavity and PhC on the light emission in the nanorod LEDs array. Near field and far-field characteristics were calculated by three dimensional finite-difference time domain (3D-FDTD) solution. It were used to analyze the modes coupling and light extraction in the nanorod LEDs arrays.

## Methods

The LED epilayer structure was grown on a c-plane sapphire substrate by metal organic chemical vapor deposition (MOCVD). It mainly consisted of a 2-μm undoped GaN layer, a 2.5-μm n-GaN layer, ten pairs of InGaN/GaN (2.2 nm/13 nm) multiple quantum wells (MQWs) with dominant wavelength at about 445 nm, and a 130 nm-thick p-GaN layer. To fabricate nanorods, a 200-nm SiO_2_ mask layer was first deposited on the LED epilayer by plasma-enhanced chemical vapor deposition (PECVD). Secondly, a 230 nm-thick resist was spin-coated on the SiO_2_ mask layer. Thirdly, the pattern of triangular nanodisks array with 380-nm diameter and 525-nm pitch were transferred to the resist layer on wafer using nanoimprint lithography (NIL) by an Obducat Eitre® 3 instrument. Next, the residual resist was removed by O_2_ plasma. And then, the exposed SiO_2_ layer was patterned by reactive ion etching (RIE) with CHF_3_ and O_2_. Finally, the GaN epilayer was etched with SiO_2_ mask using a gas mixture of Cl_2_/BCl_3_/Ar by ICP etcher. Truncated cone-shaped nanorods were formed. To remove the etching damages on the nanorod sidewalls, the etched samples were dipped into 150 °C KOH glycol solution for 30 min. The mass fraction of the KOH solution is 10 %. The n-type GaN layer was etched more quickly than the p-type GaN layer, leading to “flashlight” shaped nanorod LEDs, as mentioned in Ref. [20]. By extending the time for etching residual resist, the diameter of nano-etch mask was reduced. Then, the nanorods with the top diameter from 120 to 300 nm were obtained. And the height of the nanorods was about 1 μm.

The morphologies of these GaN-based nanorod LED samples were carefully studied by scanning electron microscopy (SEM, Nova Nano SEM 430). For TDPL measurements, samples were stuck on a copper stage cold finger in a helium closed circuit cryostat with vacuum silicone. The temperature was changed from 8 to 300 K. A 405-nm laser was used for excitation. The excitation-power density was about 100 W/cm^2^, where the carrier density in the active region was estimated as 2 × 10^18^/cm^3^. The luminescence signal was coupled into a grating spectrometer. Then the dispersed luminescence was detected by a photomultiplier tubes (PMT). To compare the PL intensities of different size nanorod LEDs, a integrating sphere was used to avoid the effect of spatial emission distribution, where the incident direction was perpendicular to the surface of the samples. The TRPL measurements were performed by LifeSpec-Red Picosecond Lifetime Spectrometer. A pulsed 372-nm laser was used as excitation source with a pulse duration of 69 ps. The laser excitation-power density was approximately 100 W/cm^2^ too. The radiation patterns of nanorod arrays were measured by ARPL. The samples were excited by a 405-nm laser with a vertical incident direction. The samples and laser spot were fixed at the rotation center of a rotary stage. The luminescence from MQWs went through a small aperture diaphragm, and then was focused into a fiber optic spectrometer. The fiber optic spectrometer was set 15 cm away from the laser spots on samples. During the measurements, the incident direction of laser was fixed, and the probed direction was changed from −90° to 90°, which corresponded to the zenith angle of the far-field pattern. The azimuth angles were set as zero according to the reference edge of the samples. At last, the full 3D-FDTD simulation was performed to illustrate the electric field distribution of a single nanorod and nanorod arrays. Perfect matched layer (PML) boundary condition was adopted. The simulation area of nanorod array was 5 × 5 μm^2^. The detector layer for far field was placed above the upper surface 300 nm. The detector layer for near field was positioned 20 nm spacing from the *c*-axis of nanorod. It is generally thought that spacing less than λ/2π is in the near field range. The far-field results were obtained by applying the Fourier transform (FT) to the near field results [27]. A single dipole source (*λ* = 400~470 nm) polarized in the in-plane direction was positioned at the center of MQWs layer, which was similar to the SpE in MQWs. The refractive indices of GaN layers, InGaN QWs layers and air were set to be 2.52, 2.58, and 1.0, respectively. The absorption coefficient of GaN and InGaN QWs were chosen to be 10 and 5000 cm^−1^.

## Results and discussion

**Fig. 1:**
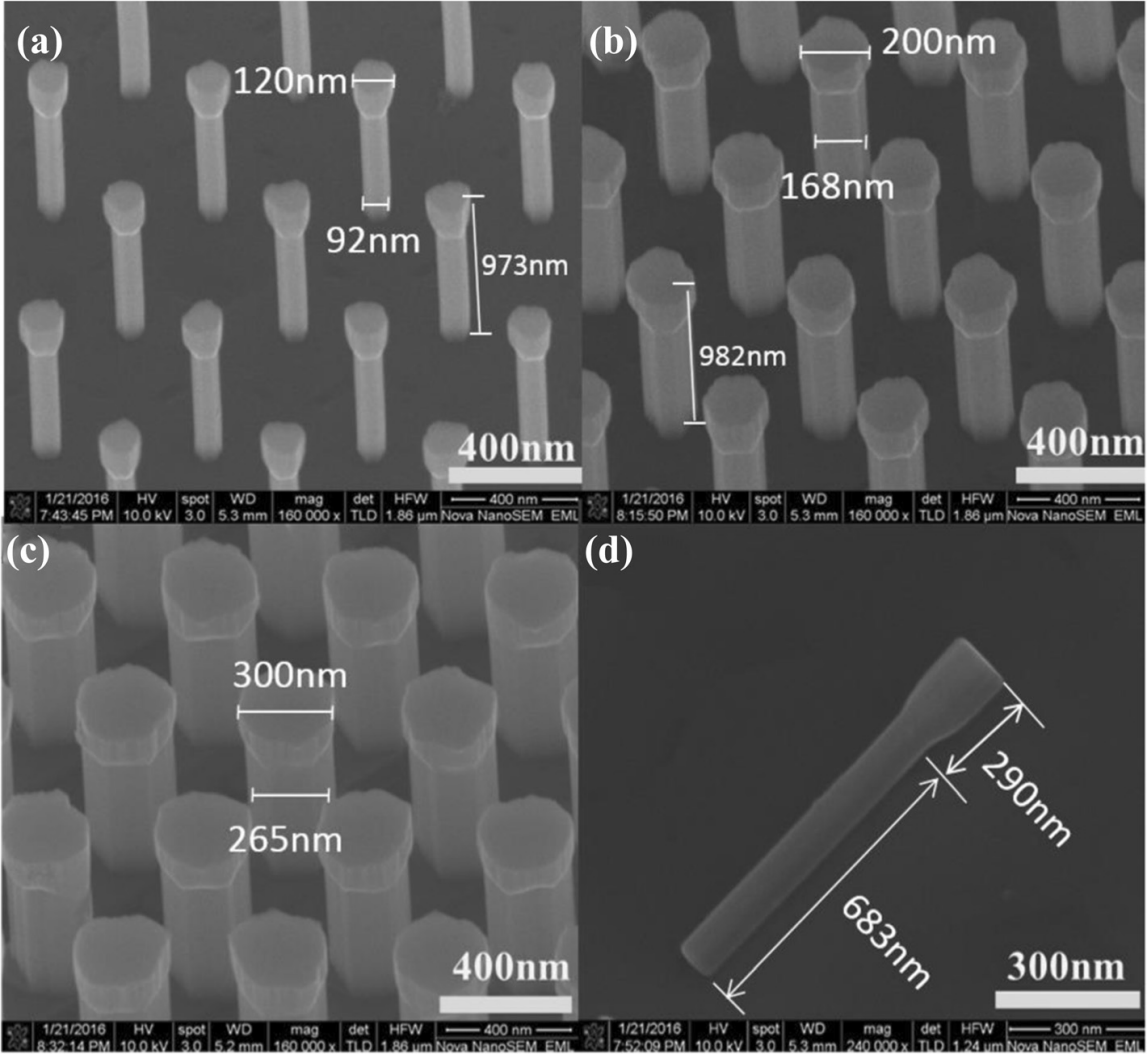
SEM images of nanorods with top diameters of **a** 120, **b** 200, and **c** 300 nm. **d** SEM image of a lying nanorod with top and bottom diameters of 120 and 92 nm, respectively

Figure 1 shows the SEM images of GaN/InGaN nanorod LED arrays with different diameters. All the nanorods show the “flashlight” shape. The top surface and sidewall are smooth and faceted. The top diameters are 120, 200, and 300 nm, while the bottom diameters are 92, 168, and 265 nm for nanorods in Fig. 1a–c, respectively. The height is about 1 μm for all of the three samples. It indicates that the size effect on the ICP etching is not significant. The surface potential of the MQWs layer drops monotonously from p-GaN to n-GaN [28], which leads to gradual neck shaped MQWs layers for these nanorods. The periphery of the top surface is irregular, which should be attributed to the SiO_2_ hard mask effect during ICP etching.

**Fig. 2:**
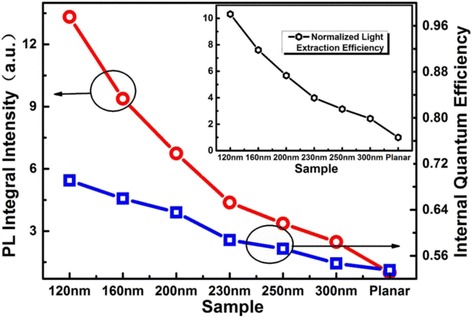
PL integral intensity per active area (*red line*) and IQE (*blue line*) for nanorod LED arrays with top diameter from 120 to 300 nm. The *inset* at top-right corner displays the normalized LEE curve by the planar sample

The PL integral intensities at room temperature for nanorod LED arrays and as-grown planar sample were measured by an integrating sphere. The results of PL integral intensities per active region area are plotted in Fig. 2, which are normalized by that of the as-grown planar sample. In Fig. 2, 13 times PL enhancement is achieved for 120 nm nanorod LED array compared with that of the planar one. With the diameter increasing, the PL enhancement is reduced. The PL enhancement is five times for 120 nm nanorod LED array compared with that of the 300 nm nanorod LED. It is reported that the junction temperature for smaller size nanorod LEDs is higher [29], which means more enhancement for 120 nm nanorod LED when the junction temperature keeps constant. Moreover, considering the gradual neck shaped active region, the PL enhancements per active area of nanorod LEDs should be higher. Here, three aspects may contribute to the high PL enhancement: laser absorption efficiency, IQE, and LEE. Because of the small filling factor and efficient absorption of InGaN MQWs for the small size nanorods array, the diffraction effect of the PhC does not enhance the absorption of laser [16, 30]. On the other hand, the incident direction of laser is nearly perpendicular to the surface of all the samples, the contribution of cavity resonance absorption and slab layer reflection can be ignored. Even if the incident direction of laser parallels to the surface of samples, the laser absorption efficiency of 120-nm nanorod LED array is estimated less than three times of that of planar one. Therefore, we mainly attribute the PL enhancement to the improvements of IQE and LEE.

To assess the improvement in IQE, TDPL measurement was carried out with the temperature ranged from 8 to 300 K. The IQE can be estimated as the ratio of the PL integral intensity at 300 K to that at 8 K. This method is based on a assumption that the IQE at low temperature is 100 %. Since all the samples is from the same epitaxial wafer and the temperature of 8 K is low enough, the method is quite appropriate to make comparison of IQE relatively. It is noticeable that the excitation-power density must be moderate for less thermal effects. The sample junction temperature must be close to the cryostat temperature as far as possible. Figure 2 shows that the IQE increases as the diameter of the nanorods decreasing. The IQE of 120-nm nanorod array LED is 69.1 %, which increased 29.1 % compared with that of planar sample. The peak wavelengths of PL spectra at 300 K for the nanorod LED arrays blue shift compared with that of the planar sample. Especially the blue shift is about 7 nm for 120 nm nanorod array. Strain relaxation of nanorods was confirmed in many reports [6–8, 10, 14, 21]. It will improve the overlaps of the electron and hole wave function, and reduce the coupling between LO-phonon and exciton [31]. So strain relaxation can improve the IQE of the nanorod LED. When the diameter is reduced to 130 nm, the strain relaxation will be saturated [32]. Moreover, the structure with smaller size is not stable enough, so the minimum diameter of the nanorod is 120 nm in this work.

The normalized LEE can be obtained by dividing the normalized PL integral intensity by the IQE. The normalized LEE curve is shown in the inset of Fig. 2. As the diameter of nanorods decreasing, the LEE increases. It reaches 10.3 and 4.5 times for 120-nm nanorod LED array compared to those of the planar and 300 nm nanorod LEDs, respectively. The enhancement of LEE may be due to the reflection of nanocavity [33], the interaction between guided modes and excitons in active region [34], and the diffraction of PhC [13–15].

**Fig. 3:**
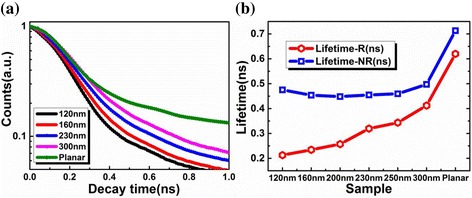
**a** TRPL decay traces of 120, 160, 230, 300 nm and planar samples at room temperature. **b** Radiative recombination lifetimes (lifetime-R) and nonradiative recombination lifetimes (lifetime-NR) for nanorod array and planar samples

TRPL spectra of nanorod LED array with different sizes have been measured at 300 K, as shown in Fig. 3a. It is observed that the decay rate increases when the size of the nanorod reduces. The PL intensity decays faster for all the nanorod samples than that for the planar one. Before the PL intensity decaying to 1/e of the maximum intensity, the carrier density in active region is close to the carrier density in TDPL experiments. A single exponential fitting can be used to get the PL lifetime (τ_PL_). τ_PL_ increases from 146 to 225 ps for nanorod array samples with the top diameter increasing from 120 to 300 nm. For planar sample, τ_PL_ is as high as 331 ps. Further, the radiative lifetime (τ_R_) and nonradiative lifetime (τ_NR_) can be estimated by the following equations [21, 35]:

1 $$ {\displaystyle {\tau}_R}(T)=\frac{{\displaystyle {\tau}_{PL}}(T)}{\eta (T)},{\displaystyle {\tau}_{NR}}(T)=\frac{{\displaystyle {\tau}_{PL}}(T)}{1-\eta (T)}. $$

where, η (T) represents IQE at a given temperature. Figure 3b shows τ_R_ and τ_NR_ of the nanorod arrays and the planar LEDs. The τ_R_ values decrease monotonously as the diameter decreasing from 300 to 120 nm. The strain relaxation and/or the interaction between guided modes and excitons in active region [34] will deduce the short lifetime. After the planar sample was etched into nanorod array, τ_NR_ reduces. The surface state of nanorods caused by large surface-to-volume ratio would led to the nonradiation recombination [30]. When the diameter of nanorods is less than 200 nm, the nonradiative recombination lifetime increases. Studies showed that strain relaxation can not only increase the overlap of electron and hole functions, but also reduce the LO-phonon-exciton coupling [31]. It would reduce the nonradiative recombination, such as the indirect Auger recombination.

**Fig. 4:**
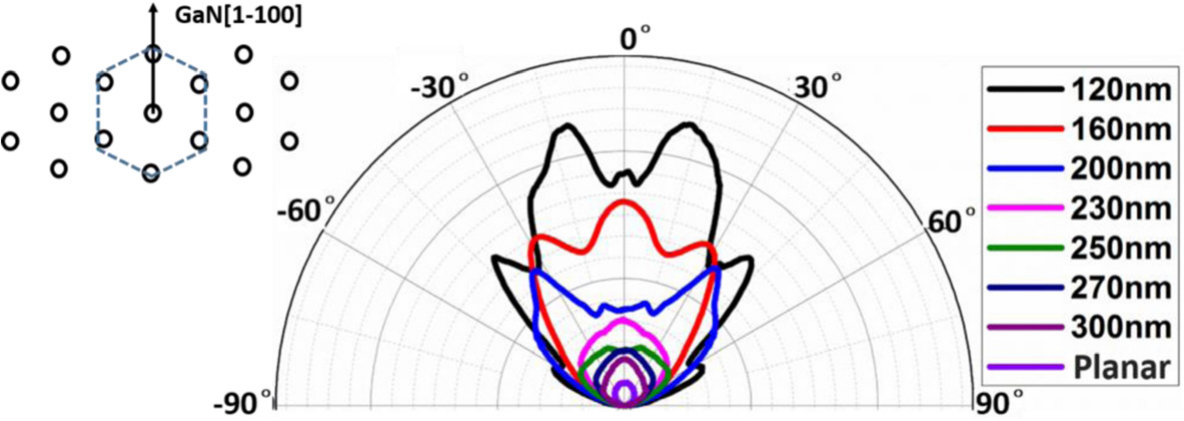
Angular distribution of PL integral intensity per active region area for nanorod array samples and planar sample in the polar coordinate. The azimuth angles were fixed at zero, it is along the direction of nanorods arrangement, which was shown in the inset

The ARPL integral intensity curves for the nanorod array samples and planar sample are shown in Fig. 4. The angular resolution is 0.5° in the zenithal direction. The planar sample shows similarly lambertian distribution with some ripples, which is caused by the Fabry–Pérot (F-P) interference. For the 300-nm nanorod sample, the far-field pattern is broadened to 60° at the half maximum, while it is about 45° for planar one. It indicates that nanorod arrays can extract out more guided modes into air [8]. With the diameter of the nanorods decreasing, the far-field pattern was strongly modified. Besides the pattern is further broadened to 68° for 250 nm nanorod sample, there are many lobes appear at 15°, 30°, and 45° and so on for smaller size nanorod samples. Rangel et al [34] shows the similar far-field pattern in their 800 nm-thick vertical structure LED. A few wave guided modes remain in such thin slab LED. When the parameters and depth of the surface patterned PhC are optimized, the preferential excitation will be coupled into these modes and extracted by the PhC. The strong coupling leads to high directionality and LEE of 73 % for the unpackaged PhC LED [34–37]. In the lateral direction of nanorod, there also exists a few guided modes as whisper gallery modes (WGMs) in nanocavity [33]. Because the structure of active region is gradient neck, the WGMs are easy to escape into the air. Besides the guided modes in the nanorods, there are also several tens guided modes in unetched n-GaN layer (slab modes) [25]. With the MQW located near the top of the nanorods, light will preferentially couple into the resonances of the nanorod, which in turn couple with these slab modes. The light in the enhanced optical modes will be extracted out by PhC. For larger size nanorod sample, there are more guided modes in the cavity, and more energy will leak into slab modes. In order to further analyze the effects of nanocavity and photonic crystal, ARPL spectra and time response ARPL were measured.

**Fig. 5:**
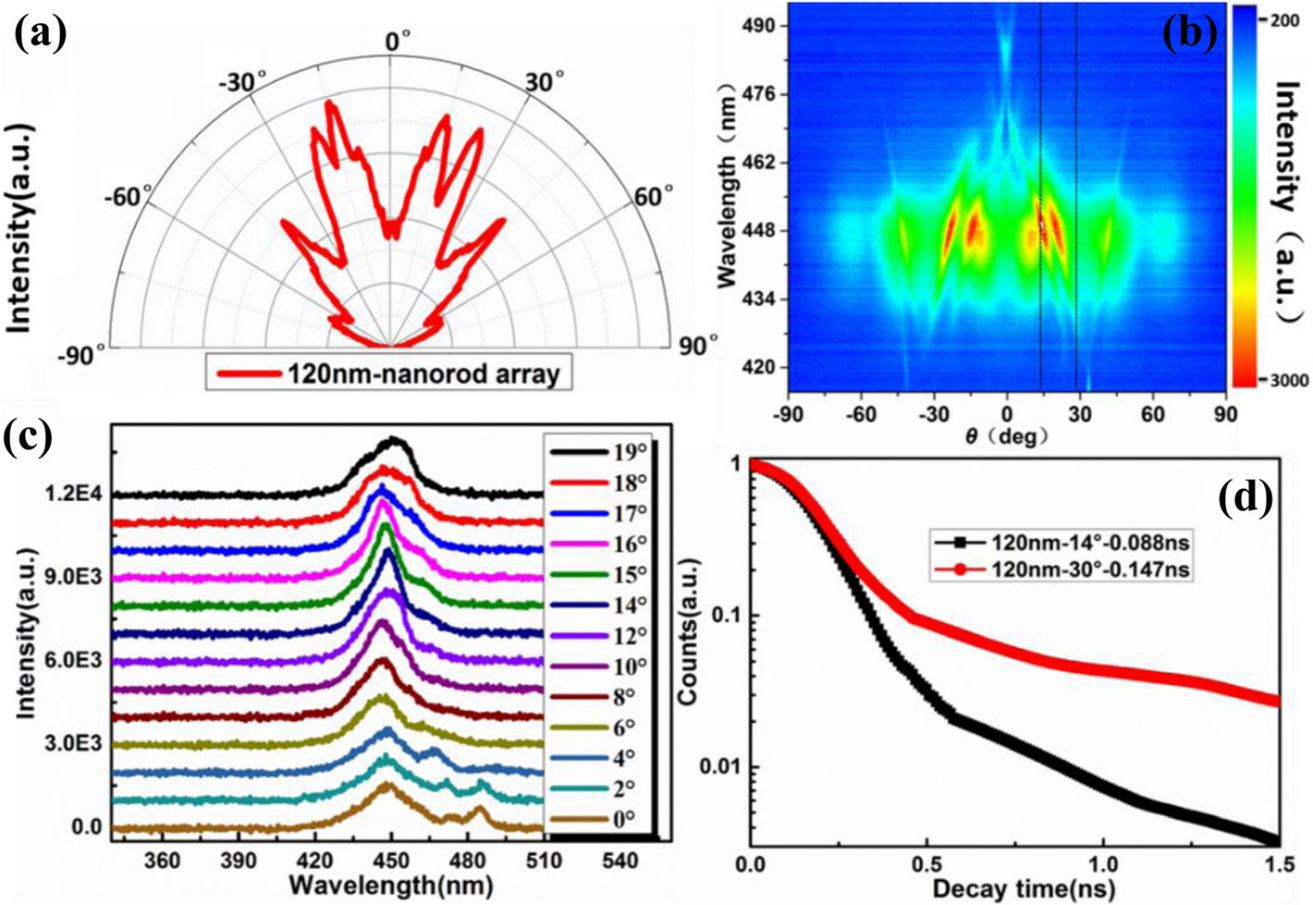
**a** Angular distribution of PL peak intensity. **b** ARPL spectra. **c** PL spectra with the zenith angles from 0° to 19°. **d** TRPL decay traces at the zenith angle of 14° and 30° for 120 nm nanorod array

Figure 5 shows ARPL results of PL spectra and time response for the 120-nm nanorod array sample. Figure 5a is angular distribution of PL peak intensity, which is sharper than that of the integral one in Fig. 4. Figure 5b shows the ARPL spectra. There are four intense bands, which are located at the zenith angle of 14°, 27°, 45°, and 68°, respectively. These intense bands correspond to specific guided modes diffracted by reciprocal lattice vectors of the PhC [38]. Less resonant bands exist in the 120-nm nanorod LED array than that in Refs. [36, 39]. It indicates that there are less guided modes in 120 nm nanocavity than that in thin film LED [36] or nanocavity above 250 nm [39]. Furthermore, most of these modes are finally diffracted into the air cone. Figure 5c shows PL spectra with zenith angle from 0° to 19°, which are involved in an intense band in Fig. 5b. The PL spectrum at 14° shows the highest peak intensity and the shallowest full width at half maximum (FWHM). The peak intensity at 14° is 2.03 times compared to that at 0°. The FWHM reduces from 30.0 to 15.2 nm when the angle increases from 0° to 14°. Therefore, the previous conclusion that the radiation at 14° is extracted from a strong-coupling mode is reasonable. Away from the intense band, the PL spectra become broader and lower. There are multiple peaks appear in the spectra with angle of 0°–4°. It may be caused by cavity-polariton dispersion [12] or whispering-gallery modes [40] in the nanocavity. Figure 5d shows TRPL decay trace at the zenith angle of 14° and 30° for the 120-nm nanorod array. The decay lifetimes are single exponentially fitted as 0.088 and 0.147 ns at the angle of 14° and 30°. It is reported that the lifetime about 1 ps in the strong-coupling regime is much shorter than the usual lifetime (in the order of ns) [12]. When it is excited non-resonantly, the lifetime will increase with orders. The resonant lifetime is much longer than 1 ps. It may be due to the excitation-power density which is lower than the threshold [40].

**Fig. 6:**
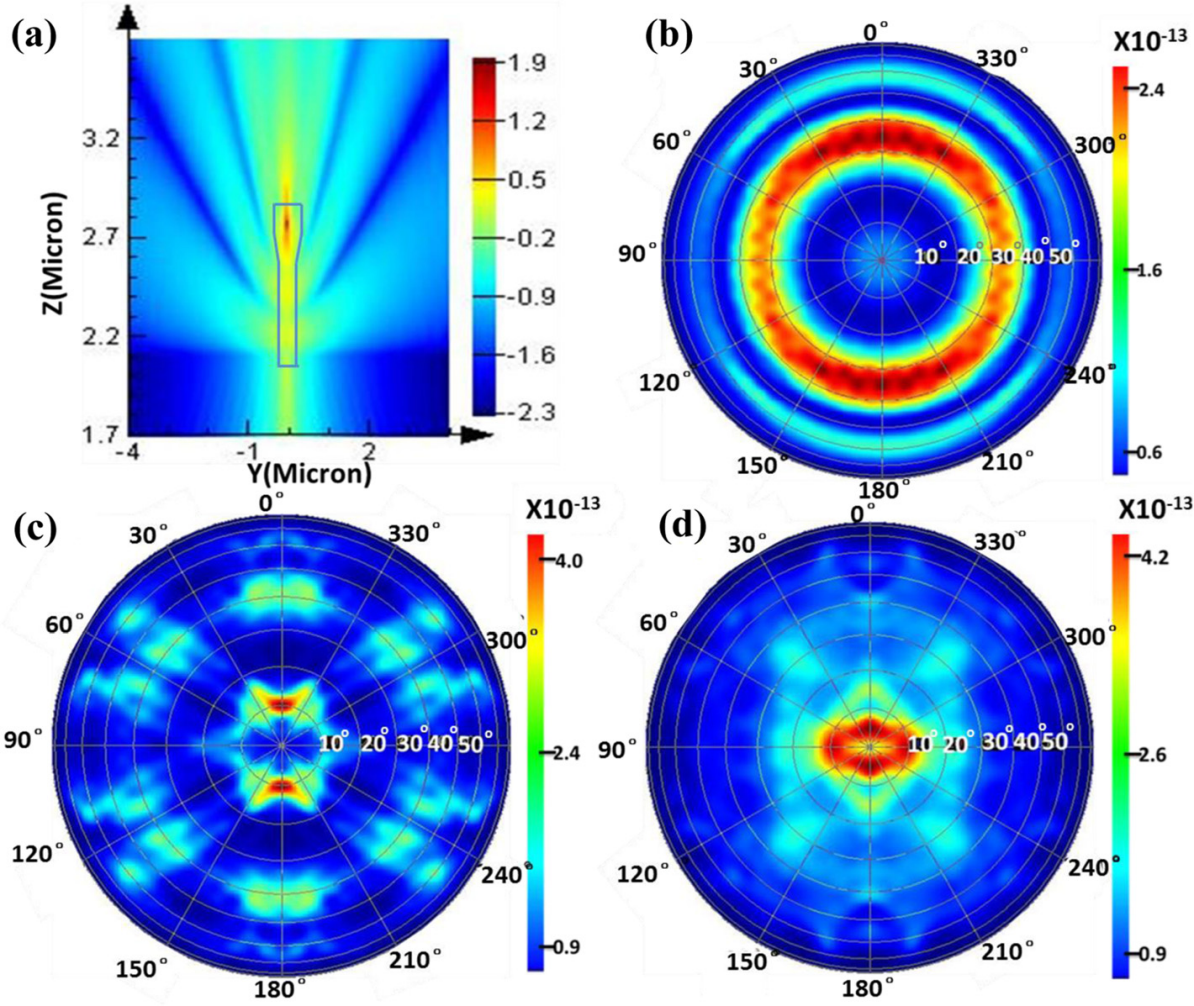
3D-FDTD simulations of **a** near field intensity distribution at 450 nm and **b** far-field radiation pattern at 450 nm for a single 120 nm nanorod LED, far-field radiation patterns at 450 nm for triangular nanorod array with diameter of **c** 120 nm and **d** 300 nm

To analyze the effects of nanocavity and PhC on light emission, full 3D-FDTD simulation was performed. Figure 6a shows the near field intensity distribution of a single 120-nm nanorod LED. It is observed that the light emitting from nanorod contains three parts, namely, the top side, the sidewall and the bottom layer. The top and side emissions are dominant in the total emissions, which is similar to the results of Ref. [13]. For the nanorod sample, light emitting into the top side becomes more directional. It should be attributed to F-P resonance, which is bounded by small top surface area. The “flashlight” shaped nanorod reduced the diameter of MQWs layer and n-GaN layer, which would reduce guided modes and enhance the light emitting out of the sidewall. Then, the light into the bottom layer would be reduced, and the slab modes in the unetched n-GaN layer would be reduced as well.

The far-field radiation pattern for a single 120-nm nanorod LED is shown in Fig. 6b. The pattern is plotted in a polar coordinates, where the polar angle corresponds to the azimuth angle, and the polar radius corresponds to the zenith angle. There are three concentric rings in the pattern, which indicate the circular symmetry and the strong coupling of guided modes. The far-field radiation patterns for 120 and 300 nm nanorod array LED samples are shown in Fig. 6c, d. A dipole source (*λ* = 400~470 nm) polarized in the in-plane direction was positioned at the center of the MQWs layer of the central nanorod. The far-field radiation pattern of 120 nm nanorods shows a hexagonal symmetry. Three broken concentric rings can be seen clearly. The change from circular symmetry in Fig. 6b to hexagonal symmetry in Fig. 6c in the far-field pattern is obvious evidence for enhanced PhC effect. The strong coupling of the resonances of individual nanorods with those of neighboring nanorods to form modes with a strong Bloch-like character that propagate laterally through the nanorod array [39, 41]. The resulting Bloch mode displays low losses in the PhC with a low effective index, in spite of the leakage in the GaN buffer and sapphire substrate. Therefore, the presence of slab modes can be neglected. The far-field intensity in Fig. 6c is basically corresponding to the results of ARPL. It is noticeable that the zenith angles of rings are 11°, 40°, and 60°, respectively. These angles are a bit less than the experimental results in Fig. 4. This can be corrected by adjusting the simulation parameters carefully. As to the far-field radiation pattern of 300 nm nanorod array, the concentric rings cannot be seen, which means weak coupling modes in the nanorod. It only displays a broad pattern, as shown in Fig. 4. Anyway, the far-field patterns of the nanorod LEDs are well simulated, and the modes coupling in nanocavity and diffracted by PhC are demonstrated.

## Conclusions

In conclusion, the “flashlight” shaped InGaN/GaN-based nanorod arrays were fabricated by nanoimprint, ICP dry etching and KOH wet etching. The light output intensity per active region area of 120-nm nanorod arrays is improved 13 times compared to that of planar LED. By TRPL and ARPL measurements, the obvious coupling of guided modes with quantized excitation is observed. FDTD simulations reveal the light extracted from coupling modes in the nanocavity by the PhC. The structures of the “flashlight” shaped nanorod and MQWs active layer can be optimized for further light emission enhancement.

## Abbreviations

3D-FDTD, three dimension finite difference time domain; ARPL, angular-resolved PL; ARPL, angular-resolved PL; F-P, Fabry–Pérot; FWHM, full width at half maximum; ICP, induced coupled plasma; IQE, internal quantum efficiency; KOH, potassium hydroxide; LED, light emitting diode; LEE, higher light extraction efficiency; lifetime-NR, nonradiative recombination lifetimes; lifetime-R, radiative recombination lifetimes; MOCVD, metal organic chemical vapor deposition; MQWs, multiple quantum wells; NIL, nanoimprint lithography; PECVD, plasma-enhanced chemical vapor deposition; PhC, photonic crystal; PL, photoluminescence; PML, perfect matched layer; PMT, photomultiplier tubes; QCSE, quantum confined Stark effect; QWs, quantum wells; RIE, reactive ion etching; SEM, scanning electron microscopy; SpE, spontaneous emission; TDPL, Temperature dependent PL; TRPL, time-resolved PL; TRPL, time-resolved PL; WGM, whisper gallery modes
